# Electrolyzed Hydrogen Water Improves Chemosensitivity to Anticancer Drugs by Potently Suppressing Autophagy

**DOI:** 10.1111/jcmm.71011

**Published:** 2026-02-17

**Authors:** Satoshi Yano, Liangjing Xie, Jinjuan Li, Yuka Sugaya, Yuki Miyauchi, Shigeru Kabayama, Taichi Hara

**Affiliations:** ^1^ Laboratory of Food and Life Science, Faculty of Human Sciences Waseda University Tokorozawa Japan; ^2^ Nihon Trim Co. Ltd. Osaka Japan

**Keywords:** anticancer drug, autophagy, chemosensitivity, hydrogen, nutrigenomics

## Abstract

Autophagy is an intracellular recycling system that contributes to the maintenance of cellular homeostasis. However, by providing tolerance to various stressors, autophagy promotes the survival and proliferation of cancer cells and confers resistance to chemotherapy. Anticancer drugs activate autophagy, leading to drug resistance in cancer cells. Autophagy inhibitors, such as chloroquine and hydroxychloroquine, enhance the antitumor effects of anticancer drugs when used in combination with them. However, these inhibitors are associated with several adverse effects. Here, using genome‐wide RNA sequencing analysis, we deciphered a novel function of electrolyzed hydrogen water (EHW) in suppressing autophagy by activating mammalian target of rapamycin complex 1 signalling. The combination of EHW with anticancer drugs, such as 5‐fluorouracil or paclitaxel, which activate autophagy, significantly decreased the viability of cervical and colorectal cancer cells. Mechanistically, molecular hydrogen and trace elements in EHW may suppress autophagy and potentiate anticancer effects. Considering its safety, we propose that EHW can act as a novel adjuvant to anticancer therapy.

## 1 Introduction

Molecular hydrogen, the smallest and lightest molecule, has unique chemical properties; it is neutral and non‐polar. Previously, it was considered nontoxic and physiologically inert because of its extremely low solubility and reactivity in pure water. However, since the 2007 report that molecular hydrogen selectively scavenges reactive oxygen species (ROS) as a potent reducing agent [1], its therapeutic effects on a variety of disease models and human diseases [2, 3], especially considering its high bioavailability via permeability across biological membranes [4, 5], have been investigated.

Electrolyzed hydrogen water (EHW), produced by electrolyzing tap water through an activated carbon filter in a TRIM ION GRACE (Nihon Trim Co. Ltd., Osaka, Japan), is free of bacterial and microscopic impurities, is rich in dissolved molecular hydrogen, contains small amounts of platinum nanoparticles derived from platinum electrodes, and is alkaline because of hydroxide ions [6, 7]. EHW prevents lifestyle‐related diseases; for example, it prevents inflammatory bowel disease [8], gastric damage [9] and immediate‐type allergies [10] via its antioxidant, anti‐inflammatory, antiapoptotic and antiallergic effects, respectively, improves insulin resistance [11], and ameliorates alcohol‐induced hepatocyte injury [12]. We demonstrated the antioxidant effects of EHW and hydrogen‐enriched water and proposed that dissolved hydrogen is the major bioactive component [12]. However, compared to hydrogen‐enriched water, EHW has a higher ROS‐scavenging capacity, which may be an EHW‐specific function [7]. However, the mechanisms of action of EHW and several issues related to its functionality remain unclear.

Autophagy, a process that degrades cellular components through the formation of autophagosomes and their fusion with lysosomes, plays an important role in maintaining cellular homeostasis [13]. The suppression of autophagy leads to the accumulation of abnormal proteins and damaged mitochondria in cells, causing various diseases [13, 14]. In contrast, autophagy plays a multifaceted role in cancer development and progression [15, 16]. Autophagy‐deficient mice with a systemic mosaic deletion of *Atg5* and liver‐specific *Atg7* ^−/−^ mice develop benign liver tumours, but such tumours do not become malignant, indicating that autophagy is also required for the malignant progression of tumours [17]. The harsh environment in dense tumours and high‐energy metabolism during proliferation might result in nutrient deprivation, requiring increased autophagy compared with that in normal cells [18, 19]. Owing to its cytoprotective function against intra‐ and extracellular stress, increased autophagy might promote proliferation and survival of cancer cells, leading to anticancer drug resistance [20].

Activation of autophagy in cancer cells plays a key role in therapeutic resistance. DNA‐damaging anticancer drugs induce cytoprotective autophagy via *TP53* in response to DNA damage [21]. Autophagy is implicated in cancer cell resistance to paclitaxel, a mitotic inhibitor [22]. Paclitaxel is one of the most effective chemotherapeutic agents for advanced cervical cancer; however, a marked increase in autophagy‐related protein levels has been observed in paclitaxel‐resistant HeLa cervical cancer cells [23]. Autophagy inhibition by 3‐methyladenine (3‐MA) enhanced the sensitivity of resistant HeLa cells to paclitaxel [23]. Antimetabolites used in cancer therapy enhance autophagy by inhibiting energy metabolism and material recycling in vivo, mimicking a state of starvation. In colorectal cancer, autophagy activation induces resistance to 5‐fluorouracil (5‐FU), which inhibits nucleic acid synthesis; however, autophagy inhibition using inhibitors such as 3‐MA and chloroquine (CQ) augmented 5‐FU chemotherapy [24, 25]. Thus, based on the premise that enhancement of autophagy by anticancer drugs contributes to cancer cell survival, leading to drug resistance, autophagy regulation has emerged as a therapeutic challenge in cancer [26].

CQ, a potent autophagy inhibitor, is being evaluated in clinical trials as an anticancer agent [27, 28]. However, because autophagy protects against acute kidney injury, a combination of chloroquine or hydroxychloroquine (HCQ) and anticancer drugs may exacerbate renal injury [29]. HCQ causes irreversible toxic retinopathy [30]. CQ and HCQ are lysosomal inhibitors that inhibit autophagic degradation. Therefore, safer autophagy inhibitors for cancer treatment are being investigated.

In this study, we examined the potential of EHW to suppress autophagy and aimed to decipher the underlying molecular mechanism. We also evaluated the therapeutic effects of anticancer drugs that activate autophagy when used together with EHW and in autophagy‐deficient (*ATG7* KO and *ATG9* KO) cells.

## 2 Materials and Methods

### 2.1 Preparation of EHW

EHW was prepared using TRIM ION GRACE (Nihon Trim Co. Ltd., Osaka, Japan) as described previously [12]. In brief, tap water was first passed through an activated charcoal filter to remove bacteria and other microscopic impurities and then subjected to electrolysis for hydrogen enrichment. The apparatus was used to generate four types of EHW (LV1–4) by varying the electric current. Filtered water (FW) that was not subjected to electrolysis was used as the control group. EHW was subjected to different treatments to obtain EHW with specific physical and chemical properties. EHW (LV4) was autoclaved twice to generate autoclaved EHW (AC). HW, with high dissolved hydrogen content, was obtained using a kit from TRIM SEVEN WATER (Nihon Trim). HW was prepared using FW or ultrapure Milli‐Q water (MQ; Merck KgaA, Darmstadt, Germany; Milli‐Q IQ‐7005) at the same hydrogen concentration as LV4. The concentration of dissolved hydrogen in the fresh EHW was measured using a flow‐cell‐type hydrogen sensor (DH‐35A, TOADKK, Tokyo, Japan). The dissolved hydrogen concentration was as follows: LV1, 780–830 ppb; LV2, 850–880 ppb; LV3, 1060–1140 ppb; LV4, 1260–1350 ppb; and HW, 1300 ppb.

### 2.2 Reagents

5‐FU was purchased from Cayman Chemical (Ann Arbor, MI, USA; 14,416) and paclitaxel was purchased from Tokyo Chemical Industry Co. Ltd. (Tokyo, Japan; P1632). 5‐FU and paclitaxel were used at 1–100 μM for 24 h. Torin 1 was procured from Merck KgaA (475991), and bafilomycin A1 was obtained from LKT Labs Inc. (St. Paul, MN, USA; B0025). Torin 1 and bafilomycin A1 were used at 1 μM and 200 nM, respectively, as positive controls for evaluating autophagy activity.

### 2.3 Cell Culture

The OUMS‐36T‐1 immortalised human embryonic fibroblast cell line was obtained from RIKEN BRC (Tsukuba, Japan). Mouse embryonic fibroblasts (MEFs) were isolated from E12.5 embryos and transformed with pEF321‐T and an SV40 large T antigen expression vector to establish immortalised cell lines [31]. HeLa, a human cervical epithelioid carcinoma cell line, and HCT116, a human colorectal cancer cell line, were obtained from ATCC (Manassas, VA, USA). The cells were cultured in DMEM (Fujifilm Wako Pure Chemical Corporation, Osaka, Japan; 044–29765) supplemented with 10% FBS (Thermo Fisher Scientific Inc., Waltham, MA, USA; 10270–106) and 1% penicillin–streptomycin (PS; Fujifilm Wako Pure Chemical Corporation; 168–23191) at 37°C in a humidified 5% CO_2_ atmosphere. For experiments, 5× DMEM was prepared by dissolving D‐MEM powder (Fujifilm Wako Pure Chemical Corporation, 049‐33561) in MQ water and diluted 1:4 with FW, EHW, AC or HW to prepare the treatment medium.

### 2.4 Establishment of Gene KO Cells Using CRISPR‐Cas9

CRISPR guide RNA (gRNA) sequences were designed to target *ATG9* (target sequence: 5′‐AGGATATTCGAGAGAAGAAG‐3′). HEK293T cells (Thermo Fisher Scientific Inc., Waltham, MA, USA) were transfected with plasmids expressing Cas9‐T2A with a single gRNA for *ATG9A*, pCMV VSV‐G and psPAX2, using FuGENE HD (Promega, Madison, WI, USA; E2311). After culturing for 48 h, the medium containing the virus particles was collected. HCT116 cells were incubated with virus‐containing medium and polybrene (8 μg/mL) for 48 h; uninfected cells were removed by puromycin treatment (5 μg/mL) and the surviving cells were cloned. The expression of ATG9A was evaluated using western blotting with anti‐ATG9A antibody (Cell Signalling Technology Inc., Beverly, MA, USA; #13509).

### 2.5 Determination of Cellular Autophagy Flux in Transgenic Cells

Cellular autophagic flux was determined as described previously [32]. HEK293FT cells were transiently cotransfected with pMRX‐IP‐GFP‐LC3‐RFP, pCG‐VSV‐G and pCG‐gag/pol using FuGENE HD. After culturing for 72 h, the medium containing the virus particles was collected. HCT116 cells were incubated with virus‐containing medium and polybrene (8 μg/mL) for 48 h, after which uninfected cells were removed by puromycin treatment (5 μg/mL). After treatment, the cells were collected, and fluorescence intensity was measured using Cellometer Vision (Nexcelom Bioscience LLC, Lawrence, MA, USA) and Spectral Cell Analyser SA3800 (Sony Biotechnology Inc., Tokyo, Japan). FCS Express4 (De Novo Software, Glendale, CA, USA) was used for quantitative analysis. The net fluorescence derived from the probe was calculated to obtain the GFP/RFP ratio as autophagy flux.

### 2.6 Fluorescence Microscopy

After treatment, HeLa GFP‐LC3‐RFP‐LC3ΔG cells and HCT116 GFP‐LC3‐RFP were fixed in 2% paraformaldehyde, followed by mounting with SlowFade Diamond Antifade Mountant (Thermo Fisher Scientific Inc.; S36963). Cellular fluorescence was visualised using a confocal laser scanning microscope (FV3000; Olympus Corporation, Tokyo, Japan) and fluorescence microscope (BZ‐X810; KEYENCE, Osaka, Japan).

### 2.7 Transcriptional Profiling via RNA Sequencing (RNA‐Seq)

OUMS‐36T‐1 cells were treated with FW or EHW (LV4) for 4 h, after which total RNA was isolated using TRIzol reagent (Thermo Fisher Scientific Inc.; 15596026), according to the manufacturer's protocol. A cDNA library was constructed using a NEB Next Ultra RNA Library Prep kit for Illumina (New England Biolabs Inc. Ipswich, MA, USA) according to the manufacturer's protocol. RNA‐seq was performed using a NovaSeq 6000 system (Novogene, Beijing, China). The reads were mapped to the reference sequences using TopHat 2. Significant genes with absolute log_2_ fold change > 0.4 and an adjusted *p* value < 10^−1^ were identified as differentially expressed genes (DEGs).

### 2.8 Quantitative PCR

OUMS‐36 T‐1 cells were seeded in a six‐well plate (1.0 × 10^5^ cells/well) and treated with FW or EHW (LV4) for 4 h. Total RNA was isolated using the ReliaPrep RNA Cell Miniprep Systems (Promega, Madison, WI, USA; Z6012), following the manufacturer's instructions. Reverse transcription of RNA to cDNA was performed using the ReverTra Ace qPCR RT Master Mix with gDNA Remover (TOYOBO, Osaka, Japan; FSQ‐301), according to the manufacturer's instructions. Briefly, the extracted RNA was incubated at 65°C for 5 min, reacted with the 4 × DN master mix at 37°C for 5 min and reverse transcribed with the 5 × RT master mix II at 37°C for 15 min, followed by 98°C for 5 min. Real‐time PCR was performed using a Thermal Cycler Dice Real Time System III (TaKaRa, Shiga, Japan) with TB Green Premix Ex Taq II (Tli RNaseH Plus) (TaKaRa, Shiga, Japan). The thermal cycling conditions involved initial denaturation at 95°C for 30 s, followed by 40 cycles of 5 s at 95°C and 30 s at 60°C. Primers for *GAPDH*, *IL11*, *TEME158*, *STC1*, *LIF*, *CHAC1*, *DDIT4* and *SESN2* were used. The mRNA levels were calculated using the ΔΔ*C*_t_ method and normalised to those of the internal control, *GAPDH*.

### 2.9 Western Blotting

Cells were lysed in Tris‐Triton buffer containing 50 mM Tris–HCl (pH 7.4), 150 mM NaCl, 1 mM ethylenediaminetetraacetic acid, 1% (v/v) Triton X‐100 and a cOmplete protease inhibitor cocktail (Merck KgaA, Darmstadt, Germany; 04693116001). The cell lysates were centrifuged at 15,000 *g* for 15 min at 4°C and the supernatants were collected. Protein concentration was determined using the Protein Assay Bicinchoninic Acid Assay Kit (Fujifilm Wako Pure Chemical Corporation; 297‐73101). Equal amounts of lysate protein were separated using SDS‐PAGE and transferred onto Mini 0.2 μm polyvinylidene difluoride transfer packs (Bio‐Rad, Hercules, CA, USA; #1704156) using a Trans‐Blot Turbo Transfer System (Bio‐Rad). The membrane was first blocked with TBST buffer (500 mM NaCl, 20 mM Tris–HCl pH 7.4 and 0.1% Tween 20) containing 5% w/v nonfat dry milk and then incubated overnight at 4°C with specific primary antibodies (1:1000 dilution), followed by horseradish peroxidase‐conjugated secondary antibodies (1:10,000 dilution) for another 1 h. Bound antibodies were detected using the ECL system, and the relative amounts of proteins associated with specific antibodies were quantified using FUSION SOLO S (Vilber Lourmat, Marne‐la‐Vallée, France). The antibodies used for western blotting, Phosho‐p70 S6K (#97596) and LC3A/B (#12741), were purchased from Cell Signalling Technology Inc. β‐Actin (MAB1501) was purchased from Millipore Inc. (Billerica, MA, USA). Horseradish peroxidase‐conjugated mouse and rabbit IgGs (115–035‐003 and 111‐035‐144, respectively) were purchased from Jackson ImmunoResearch Laboratories (West Grove, PA, USA).

### 2.10 Cell Viability Assay

Cell viability was measured using Cell Counting Kit‐8 (Dojindo, Kumamoto, Japan; CK04). Cells (1.0 × 10^4^ cells/well) were seeded into 96‐well plates and treated with or without anticancer drugs for 24 h. Cell Counting Kit‐8 solution was added to each well, and incubation was continued for another 1 h. The intensity of the yellow‐coloured formazan dye was determined by measuring the absorbance at 450 nm using a Multiskan FC microplate photometer (Thermo Fisher Scientific). Cell viability was expressed as a ratio of the optical density for the treatment to that for the control.

### 2.11 Statistical Analysis

Data represent the mean ± standard error of the mean (SEM). Statistical analyses were performed using a two‐tailed Student's *t*‐test and one‐way ANOVA for multiple comparisons. *p < 0.05, **p < 0.01 and ***p < 0.001 were considered to indicate significant differences. Different letters above the bar indicate significant differences (*p* < 0.05).

## 3 Results

### 3.1 Transcriptome Profiling Suggests That EHW Activates mTORC1 Signalling

**FIGURE 1:**
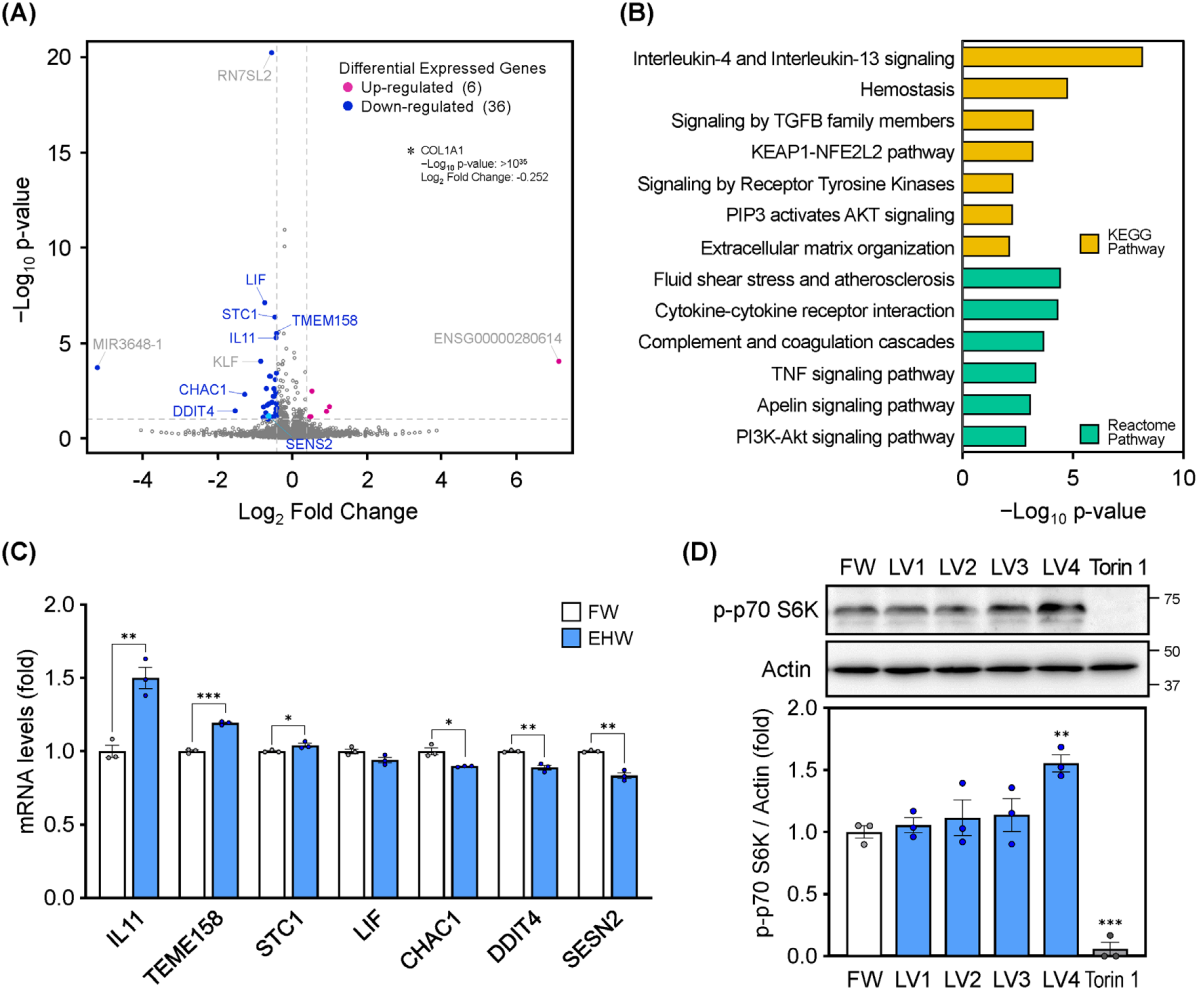
Electrolyzed hydrogen water (EHW) activates the PI3K/Akt/mTOR pathway, as evidenced by transcriptome profiling using RNA‐seq. (A) Volcano plot showing the differential expression of genes in EHW versus filtered water (FW) treatment. The x‐ and y‐axes present log_2_ fold change of EHW/FW and −log_10_ *p* value, respectively. Differentially expressed genes (DEGs) were determined based on differential expression thresholds (*p* value < 0.1 and |log_2_ fold change|> 0.4). (B) Analysis of the biological functions of DEGs based on the Kyoto Encyclopedia of Genes and Genomes (KEGG) and Reactome pathway database using Metascape. (C) mRNA levels of DEGs involved in the regulation of the PI3K/Akt/mTOR pathway. (D) Protein levels of phospho‐p70 S6K. OUMS‐36T‐1 cells were treated with FW or EHW for 4 h. Whole cell lysates were used for analysis. Data are presented as the mean ± SEM (*n* = 3). Asterisks indicate significant differences determined using the Student's *t*‐test. *p < 0.05, **p < 0.01, ***p < 0.001 versus FW.

To comprehensively analyse the function of EHW, we assessed its effect on the transcriptome. A total of 42 differentially expressed genes (DEGs) were identified; 6 genes were upregulated and 36 were downregulated in the EHW treatment compared to the FW treatment (Figure 1A). The biological functions of the extracted DEGs were analysed using the Kyoto Encyclopedia of Genes and Genomes and Reactome databases. The DEGs were characteristically enriched for cytokine‐related functions (interleukin‐4 and interleukin‐13 signalling, signalling by TGFB family members, cytokine‐cytokine receptor interaction and TNF signalling pathway) and functions related to kinases involved in survival and proliferation (signalling by receptor tyrosine kinases, PIP3 activates Akt signalling and PI3K‐Akt signalling pathway) (Figure 1B). As the PI3K/Akt/mTOR pathway is also implicated in immunomodulation, DEGs related to the regulation of the PI3K/Akt/mTOR pathway were analysed using qPCR. Expression of *IL11* [33], *TMEM 158* [34] and *STC1* [35], which activate the PI3K/Akt/mTOR pathway, was significantly increased, whereas that of *DDIT4* [36] and *SESN2* [37] was significantly decreased following EHW treatment (Figure 1C). p70 S6K is downstream of mTORC1 signalling and reflects its activity. A significant increase in p70 S6K phosphorylation levels was observed following EHW LV4 treatment (Figure 1D).

### 3.2 EHW Has an Inhibitory Effect on Autophagy

**FIGURE 2:**
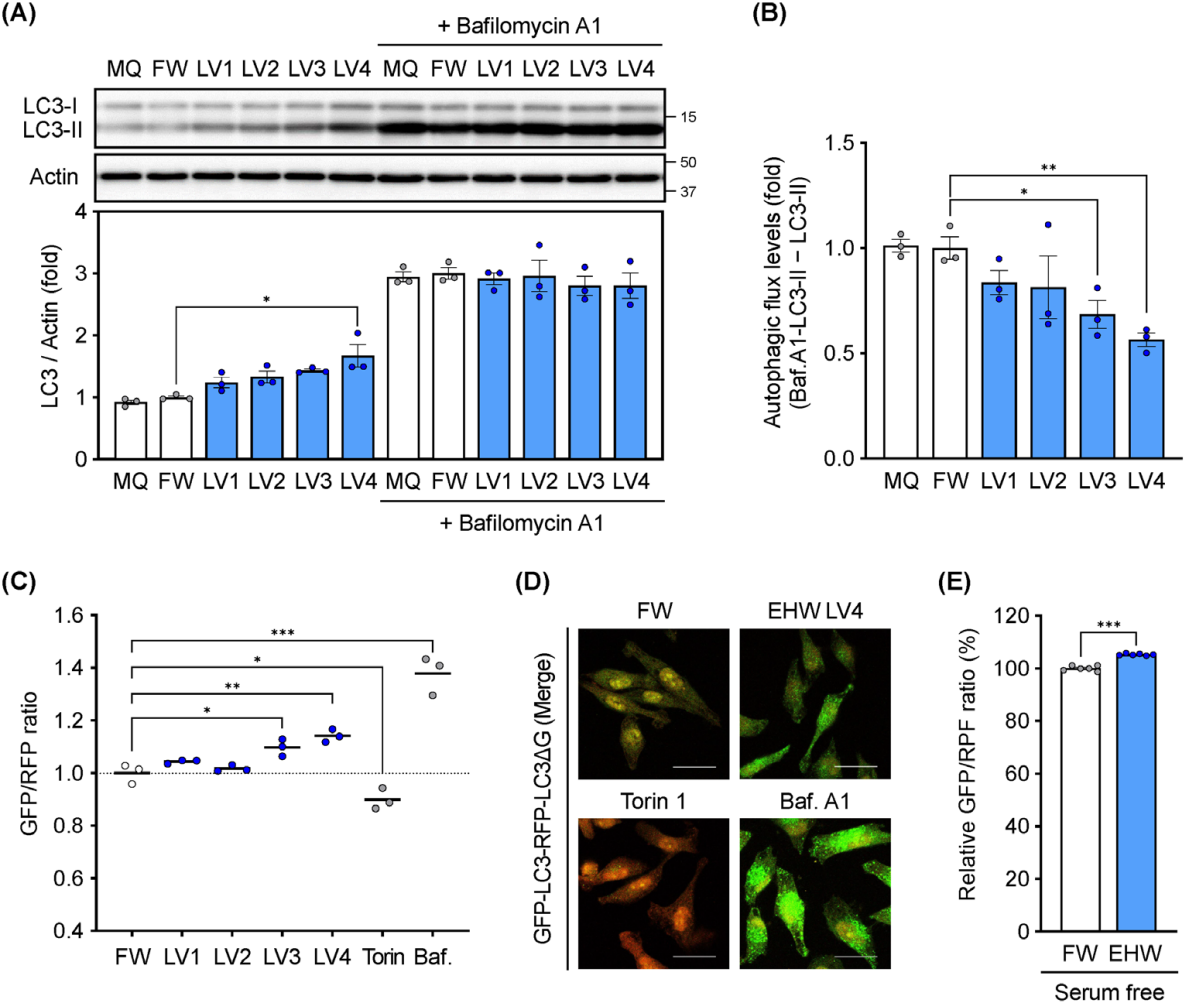
Electrolyzed hydrogen water (EHW) regulates the autophagy flux. (A) LC3 turnover assay. Mouse embryonic fibroblasts (MEFs) were treated with ultrapure Milli‐Q water (MQ), filtered water (FW) or EHW (LV1–LV4) for 4 h. Whole cell lysates were used for western blot analysis using an LC3‐specific antibody. (B) Autophagy flux was obtained by subtracting LC3‐II levels from LC3‐II levels with bafilomycin A1 based on (A). (C) Effect of EHW on autophagy flux and (D) fluorescence microscopy imaging. HeLa GFP‐LC3‐RFP‐LC3ΔG cells were treated with FW, EHW (LV1–4), 1 μM torin 1 or 200 nM bafilomycin A1 (Baf.) for 4 h. The intensity of the fluorescent probe was detected using Cellometer Vision (C) or Spectral Cell Analyser SA3800 (D). (E) Effect of EHW on autophagy flux under serum‐free conditions. Data are presented as the mean ± SEM (*n* = 3 or 6). Asterisks indicate significant differences determined using the Student's *t*‐test. *p < 0.05, **p < 0.01, ***p < 0.001 versus FW.

EHW activated mTORC1 signalling (Figure 1) and may therefore suppress autophagic activity. We quantitatively analysed the effect of EHW on autophagy using an LC3 turnover assay with bafilomycin A1. EHW (LV4) treatment increased the levels of LC3‐II; however, no significant difference was observed between the FW and EHW groups in the bafilomycin A1‐treated group (Figure 2A). Analysis of the difference in LC3‐II levels as an indicator of autophagy flux between the bafilomycin A1‐treated and untreated groups showed a level‐dependent decrease in autophagy flux following EHW treatment (Figure 2B). Analysis of autophagy flux based on a newly established method using a GFP‐LC3‐RFP‐LC3ΔG probe [38] revealed that the GFP/RFP ratio was inversely correlated with the autophagic activity of the cells. Torin 1 (an autophagy activator) decreased the GFP/RFP ratio, and bafilomycin A1 (an autophagy inhibitor) increased it (Figure 2C). EHW (LV3 and LV4) treatment significantly increased the GFP/RFP ratio. Fluorescence imaging also revealed a stronger green fluorescence intensity in the EHW treatment than in the FW treatment (Figure 2D). During starvation‐induced autophagy, mTORC1 signalling is suppressed. EHW activated mTORC1 signalling (Figure 1D) and significantly suppressed activation of autophagy under serum‐free conditions (Figure 2E).

### 3.3 EHW Improves the Anticancer Effect of Paclitaxel by Inhibiting Autophagy Activation

**FIGURE 3:**
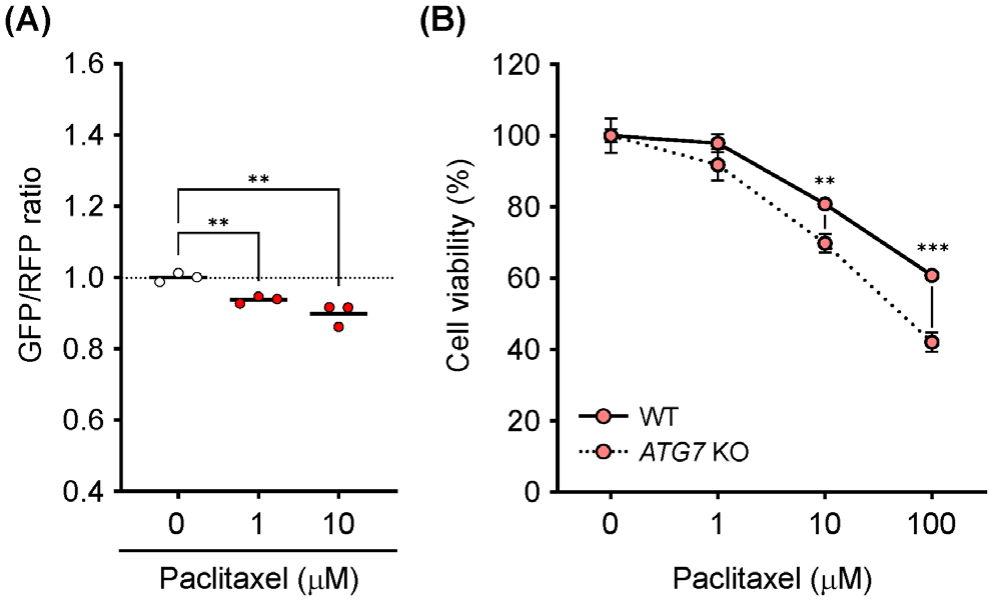
Effects of anticancer drugs on autophagy and cell viability. (A) Autophagy flux analysis based on the GFP/RFP ratio. HeLa cells expressing GFP ‐LC3‐RFP‐LC3ΔG were treated with paclitaxel (0–10 μM) for 4 h. After treatment, the cells were collected, and the fluorescence intensity was measured using the Spectral Cell Analyser SA3800. (B) Cell viability. HeLa cells were treated with paclitaxel (0–100 μM) for 24 h. The cell viability was measured using the Cell Counting Kit‐8. Data are presented as the mean ± SEM (*n* = 3 or 6). Asterisks indicate significant differences determined using the Student's *t*‐test. **p < 0.01, ***p < 0.001 versus FW.

**FIGURE 4:**
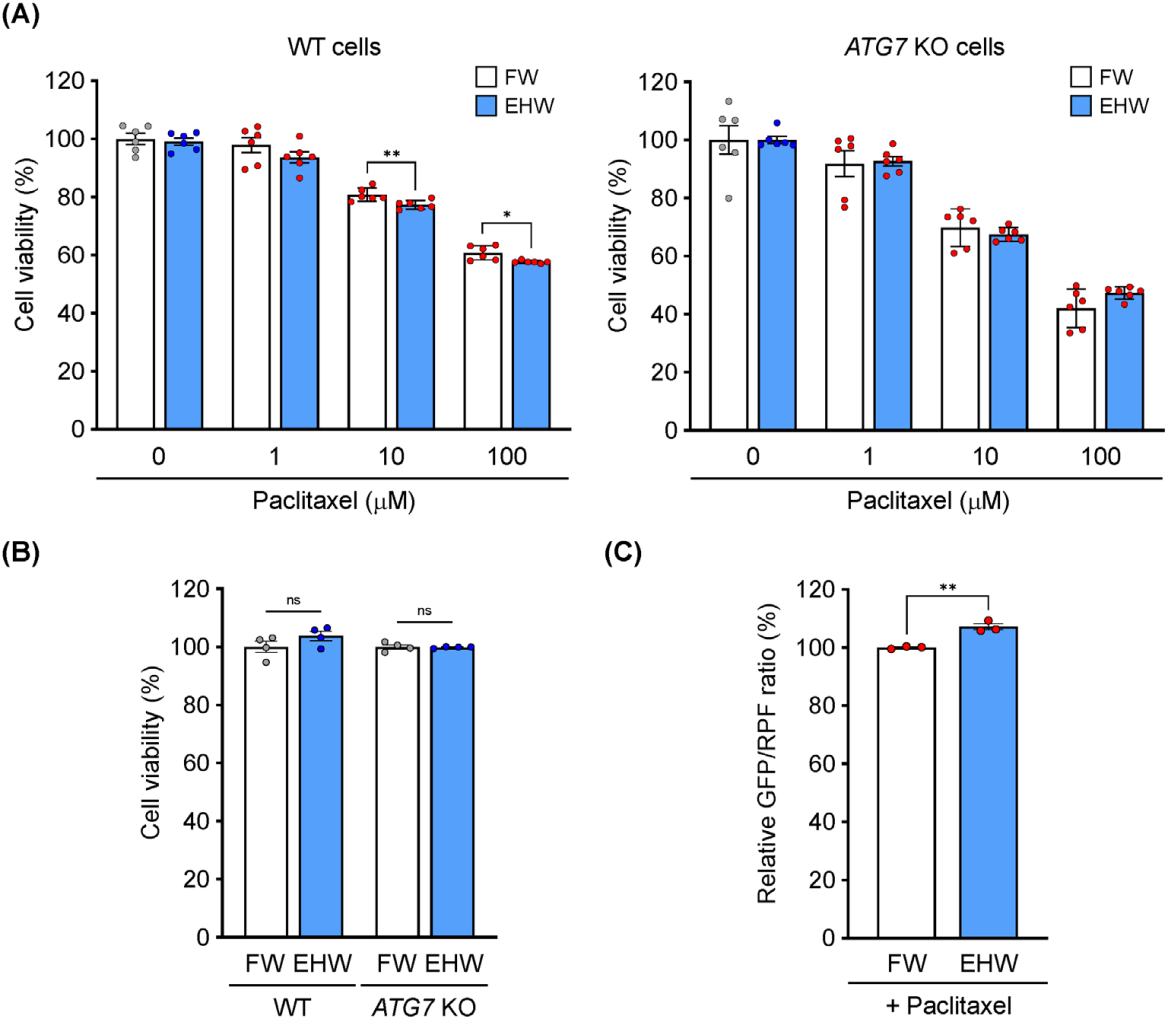
Electrolyzed hydrogen water (EHW) improves the anticancer effect of paclitaxel via autophagy inhibition. (A) Cell viability. HeLa and HeLa *ATG7* KO cells were treated with paclitaxel (0–100 μM) in a medium prepared using filtered water (FW) or EHW for 24 h. The cell viability was measured using Cell Counting Kit‐8. (B) Effect of EHW on cell viability. (C) Autophagy flux analysis based on the GFP/RFP ratio. HeLa cells expressing GFP‐LC3‐RFP‐LC3ΔG were treated with paclitaxel (10 μM) in a medium prepared using FW or EHW for 4 h. After treatment, the cells were collected, and fluorescence intensity was detected using Spectral Cell Analyser SA3800. Data are presented as the mean ± SEM (*n* = 3–6). Asterisks indicate significant differences determined using the Student's *t*‐test. *p < 0.05, **p < 0.01 versus FW.

Because EHW suppressed autophagy in HeLa cells (Figure 2C,D), we investigated whether it improved the anticancer effect of paclitaxel. Paclitaxel significantly activated autophagy in a concentration‐dependent manner (Figure 3A). Paclitaxel was more effective in suppressing cell viability in autophagy‐deficient (*ATG7* KO) cells than in wild‐type cells (Figure 3B), consistent with previous findings that the autophagy‐activating effect of paclitaxel contributes to the survival of cancer cells. As shown in Figure 4A, 10 and 100 μM paclitaxel in combination with EHW significantly decreased the viability of wild‐type cells compared to that with FW treatment. In contrast, no significant differences were observed in the *ATG7* KO cells treated with FW and EHW (Figure 4A). EHW‐alone treatment did not affect the viability of either wild‐type or *ATG7* KO HeLa cells (Figure 4B). Furthermore, EHW significantly suppressed paclitaxel‐induced autophagy (Figure 4C).

### 3.4 EHW Improves the Anticancer Effect of 5‐FU by Inhibiting Autophagy Activation

**FIGURE 5:**
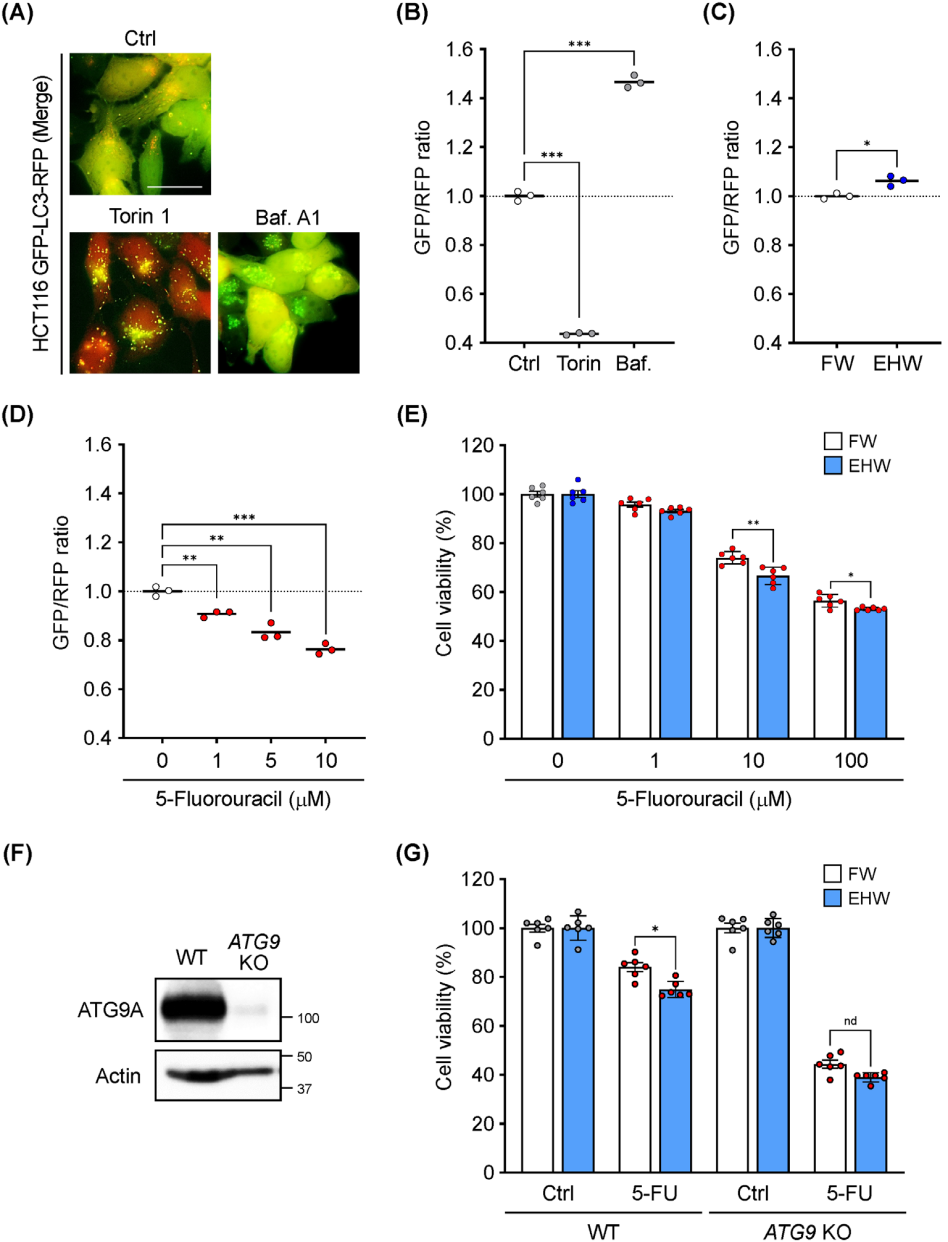
Electrolyzed hydrogen water (EHW) improves the anticancer effect of 5‐fluorouracil (5‐FU) in colon cancer cells. (A–D) Autophagy flux analysis based on the GFP/RFP ratio. HCT116 cells expressing GFP‐LC3‐RFP were treated with 1 μM torin 1 or 200 nM bafilomycin A1 (Baf.) (A, B), FW and EHW (LV4) (C), and the indicated concentrations of 5‐FU for 24 h (D). The fluorescence intensity of the probe was detected using Spectral Cell Analyser SA3800. (E) Cell viability. HCT116 cells were treated with 5‐FU (0–10 μM) in a medium prepared using filtered water (FW) or EHW for 24 h. (F) Establishment of HCT116 *ATG9* KO cells. (G) Cell viability. HCT116 cells and HCT116 *ATG9* KO cells were treated with 5‐FU (10 μM) in a medium prepared using FW or EHW for 24 h. The cell viability was measured using Cell Counting Kit‐8. Data are presented as the mean ± SEM (*n* = 3–6). Asterisks indicate significant differences determined using the Student's *t*‐test. *p < 0.05, **p < 0.01, ***p < 0.001 versus FW.

Because EHW exerts the beneficial effects of EHW on the intestine, we examined its potential for treating colorectal cancer. First, to analyse autophagy flux in human colorectal cancer cells, we established an HCT116 cell line that stably expressed the GFP‐LC3‐RFP probe. As in HeLa cells (Figure 2C,D), torin 1 decreased the GFP/RFP ratio, and bafilomycin A1 increased it, consistent with the fluoroimaging results (Figure 5A,B). EHW (LV4) treatment significantly increased the GFP/RFP ratio (Figure 5C) in HCT116 cells. In contrast, 5‐FU, an anticancer drug used for colorectal cancer, caused a concentration‐dependent decrease in the GFP/RFP ratio and activated autophagy (Figure 5D). As shown in Figure 5E, 1 μM 5‐FU in combination with EHW significantly decreased the viability of HCT116 cells compared with that of FW. Next, we generated HCT116 cells deficient in *ATG9*, an autophagy‐related gene (Figure 5F). The combination of 5‐FU and EHW significantly decreased the viability of HCT116 cells but not of *ATG9* KO cells (Figure 5G).

### 3.5 Molecular Hydrogen Dissolved in EHW Is the Major Bioactive Component That Suppresses Autophagy and Enhances Anticancer Effect

**FIGURE 6:**
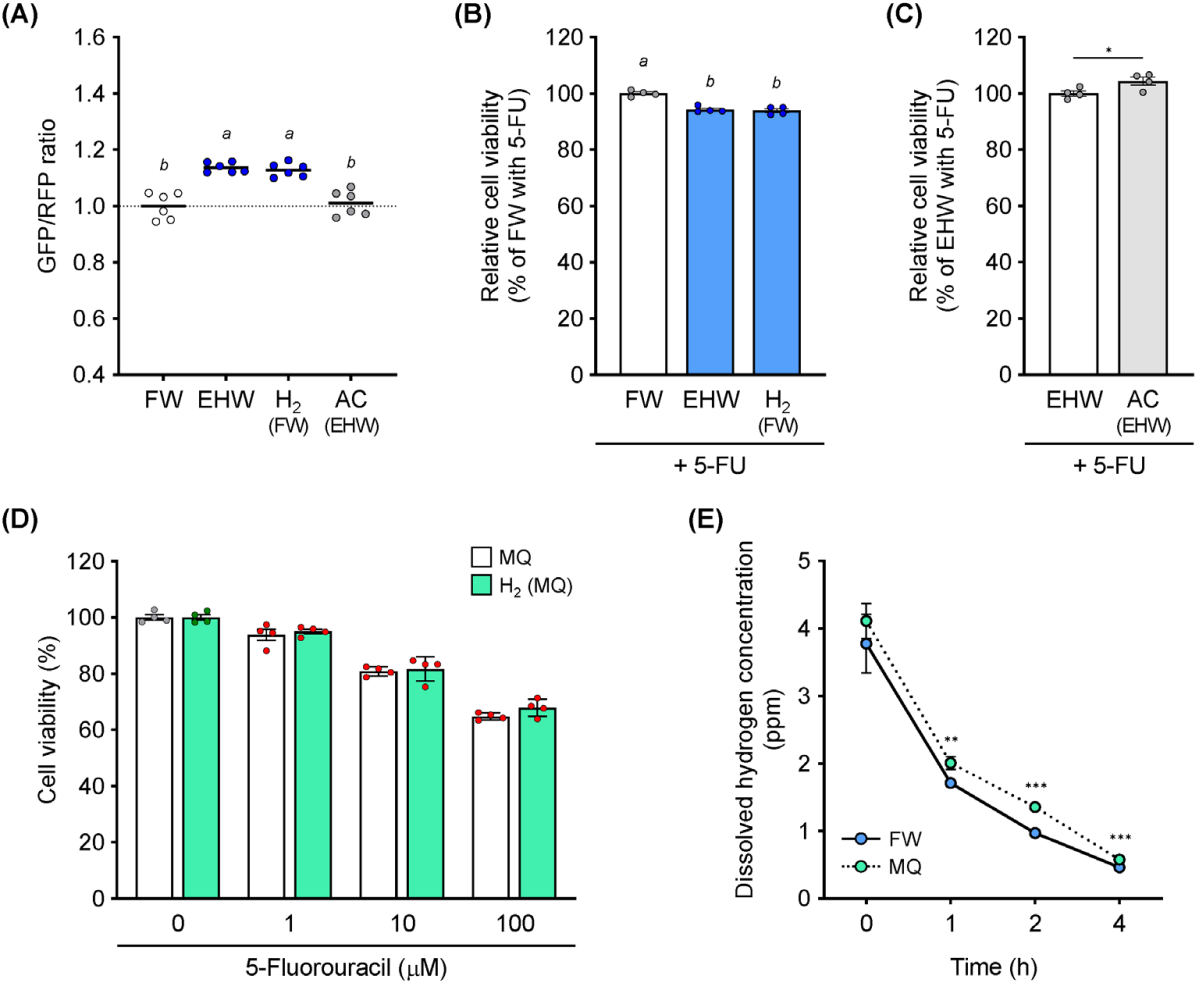
Verification of bioactive molecules in electrolyzed hydrogen water (HW) (EHW). (A) Autophagy flux analysis based on the GFP/RFP ratio. HCT116 cells expressing GFP‐LC3‐RFP were treated with filtered water (FW), EHW, HW and autoclaved EHW (AC) for 4 h. The fluorescence intensity of the probe was detected using Spectral Cell Analyser SA3800. (B–D) Cell viability. HCT116 cells were treated with 5‐fluorouracil (5‐FU; 10 μM) added to the medium prepared in FW, EHW, HW, AC, ultrapure Milli‐Q water (MQ) and HW prepared from MQ (HW(MQ)) for 24 h. Cell viability was measured using Cell Counting Kit‐8. (E) Dissolved hydrogen concentration at different time points. Data are presented as the mean ± SEM (*n* = 3–6). Asterisks indicate significant differences determined using the Student's *t*‐test. *p < 0.05, **p < 0.01, ***p < 0.001 versus FW. Different letters above the bars indicate significant differences among the groups analysed using Tukey's test after one‐way analysis of variance (*p* < 0.05).

As shown in Figure 6A, the increase in the GFP/RFP ratio exhibited by EHW was similar to that for hydrogen dissolved in FW. HW also improved the anticancer effect of 5‐FU, similar to that of EHW (Figure 6B). In contrast, the loss of this effect was observed for AC (Figure 6A,C). Thus, dissolved molecular hydrogen may contribute to the enhanced anticancer effects. However, hydrogen dissolved in MQ, not in FW, did not affect the inhibition of cell viability by 5‐FU (Figure 6D). The dissolved hydrogen concentration was significantly higher in MQ than in FW (Figure 6E).

## 4 Discussion

Genome‐wide RNA‐seq analysis revealed that EHW potently activates the PI3K/AKT/mTOR signalling. Furthermore, we identified a novel feature of EHW: its ability to suppress autophagy. In cervical (HeLa) and colorectal (HCT116) cancer cells, EHW potentiated the anticancer effects of paclitaxel and 5‐FU by suppressing their activation of autophagy. The major bioactive component in EHW might be molecular hydrogen, and other trace elements present in EHW might also play a crucial role in enhancing the anticancer effect. These results suggest that EHW might work as an adjuvant for anticancer drugs that activate autophagy.

The effect of EHW on gene expression had not been investigated previously. Based on a comprehensive analysis of the biological functions of DEGs identified using genome‐wide RNA‐seq, we found that EHW has anti‐inflammatory and antioxidant effects (Figure 1B), which is consistent with previous findings [39]. PI3K/AKT/mTOR signalling, a regulator of numerous cellular processes, was an enriched biological function term, and mTORC1 signalling activation and an increase in the phosphorylation of p70 S6K were found to be novel functions of EHW (Figure 1D). The mammalian target of rapamycin (mTOR), a phosphatase, plays a central role in the regulation of cell growth, survival, motility and morphology by sensing intracellular energy levels [40]. mTOR, which plays a role in protein synthesis, is involved in the resistance exercise‐induced increase in the synthesis of muscle protein [41], and p70 S6K, a protein downstream of mTOR, is essential for maintaining muscle function and force production [42]. Recently, EHW was reported to significantly reduce energy expenditure during endurance exercise [43], highlighting its involvement in energy metabolism and maintenance of muscle strength. The finding that EHW activates mTORC1 signalling might lead to novel insights into energy metabolism and exercise research.

EHW potentiated the therapeutic effects of the anticancer drugs (Figures 4 and 5). Upon nutrient starvation, intracellular mTORC1 signalling, which senses growth factors and amino acids, is suppressed, leading to induced autophagy in vitro and in vivo [44]. Using several approaches, we demonstrated that EHW potently activates mTOR signalling, which may contribute to the suppression of autophagy (Figure 2). Although mTOR is a key driver of drug resistance in cancer therapy, it was recently demonstrated to suppress autophagy, resulting in metabolic vulnerability related to energy crisis and apoptosis of cancer cells to anticancer drugs [45]. Verification using autophagy‐deficient *ATG7* and *ATG9* KO cells showed that EHW, an mTOR signalling activator, suppressed autophagy induced by paclitaxel and 5‐FU, potentiating their anticancer effects (Figures 4A and 5G). The combination of an anticancer drug with an autophagy inhibitor augments the therapeutic effect by improving the drug sensitivity of cancer cells [46]. Notably, cancer cells tend to be more dependent on autophagy than normal cells to maintain rapid proliferation and survival under metabolic stress [47]. Therefore, autophagy inhibition is expected to exert a more pronounced cytotoxic effect on cancer cells than on normal cells. However, autophagy inhibition is detrimental to health because of its cytoprotective effects against multiple stresses. The combination of CQ or HCQ with anticancer drugs may exacerbate renal injury because autophagy plays a protective role in acute kidney injury [29]. In contrast, EHW can reduce oxidative stress during haemodialysis in patients undergoing chronic dialysis without reported adverse events [48, 49]. Recently, an observational study in healthy adults revealed that blood urea nitrogen levels, an indicator of renal dysfunction, were significantly lower in the group with a habit of consuming EHW [50]. Furthermore, in our study, EHW treatment alone did not compromise the viability of HeLa cells (Figure 4B), nor did it induce cytotoxicity in fibroblasts (OUMS‐36 T‐1 and MEFs cells) used for mechanistic analysis. Thus, EHW has a favourable safety profile and can be used as a therapeutic adjuvant against cancer cells.

EHW is rich in dissolved molecular hydrogen and contains a small number of platinum nanoparticles. Both EHW and hydrogen‐enriched water reduce alcohol‐induced hepatocyte injury, indicating that dissolved molecular hydrogen is the major bioactive component of EHW [12]. However, EHW also has a higher scavenging capacity for ROS, which may be a function specific to EHW [7]. In this study, the suppressive effect of EHW on autophagy and its augmentation of the effects of anticancer drugs were lost upon autoclaving it (Figure 6A–C). Furthermore, hydrogen‐dissolved water in FW suppressed autophagy and augmented the anticancer effect, similar to that of EHW (Figure 6A,B), indicating that molecular hydrogen might be the major bioactive component of EHW. The combination of HW and 5‐FU promotes the induction of apoptosis in colon cancer cells [51], and hydrogen gas inhibits cancer cell growth [52, 53, 54]. Notably, HW prepared using MQ did not exhibit any of the effects of EHWor HW prepared using FW (Figure 6D). Although the physical stability, indicated by the rate of hydrogen loss, was similar between FW and MQ (Figure 6E), the biological effects were strikingly different. This result provides compelling evidence that the manifestation of EHW's biological function of EHW depends not only on the retention of hydrogen gas but also on the presence of co‐existing mineral components in the solvent. Several studies on the antioxidant activity of HW have reported that trace elements, such as vanadium, might be involved in some of its functions [55]. Previous reports have also indicated that EHW contains platinum nanoparticles derived from electrodes [6]. Consequently, these trace elements or nanoparticles may act as essential cofactors or catalysts that facilitate the interaction of molecular hydrogen with cellular targets upstream of mTORC1. Trace amounts of minerals in FW might play an important role in mediating the effects of molecular hydrogen.

Regarding the duration of hydrogen exposure, the concentration of dissolved hydrogen in the culture medium decreased over time in an open system (Figure 6E). However, we observed significant mTORC1 activation and autophagy modulation after 4 h of treatment (Figures 1D and 2). This suggests that a sustained high concentration of hydrogen for 24 h is not strictly necessary. Instead, the initial exposure to EHW likely acts as a ‘trigger’ that activates on the mTORC1 signalling pathway, leading to a sustained biological effect even after the hydrogen concentration has diminished.

In the present study, we identified novel functions of EHW, namely the activation of mTORC1 signalling and suppression of autophagy. EHW augmented the anticancer effects of paclitaxel and 5‐FU in cervical and colorectal cancer cell lines, highlighting its potential as an adjuvant for anticancer therapy. The active components of EHW, other than molecular hydrogen and the detailed mechanism of autophagy suppression remain to be elucidated. Further evidence is required to expand the scope of research on EHW in different target cancers and anticancer agents.

## Author Contributions

**Satoshi Yano:** data curation, formal analysis, investigation, methodology, supervision, visualization, writing – original draft, writing – review and editing. **Liangjing Xie:** data curation, formal analysis, investigation, validation, writing – original draft. **Jinjuan Li:** data curation, investigation, methodology, validation, writing – review and editing. **Yuka Sugaya:** data curation, investigation, methodology, validation, writing – review and editing. **Yuki Miyauchi:** supervision, visualization, writing – original draft. **Shigeru Kabayama:** resources, supervision, writing – review and editing. **Taichi Hara:** conceptualization, project administration, supervision, writing – review and editing.

## Funding

This work was supported by grants from JSPS KAKENHI, Japan (22K17816 and 24K20683 to YS and 20H03408 to HT), a Grant‐in‐Aid for JSPS Fellows, Japan (24KF0073 to HT), Waseda University Grant for Special Research Project (2023C‐214 and 2024C‐554 to YS and 2023C‐211 and 2024C‐217 to HT), and a joint research fund from Nihon Trim Co. Ltd. (to HT).

## Conflicts of Interest

K.S. is affiliated with Nihon Trim Co. Ltd. Y.S., X.L., L.J., S.Y., M.Y. and H.T. have no conflicts of interest. The sponsors played no role in the design, execution, interpretation or writing of the study.

## Data Availability

The data that support the findings of this study are available from the corresponding author upon reasonable request.
